# Entrepreneurship education of college students and entrepreneurial psychology of new entrepreneurs under causal attribution theory

**DOI:** 10.3389/fpsyg.2022.943779

**Published:** 2022-11-03

**Authors:** Shuming Xie, Jie Luo, Yixin Zheng, Chongyang Ma

**Affiliations:** ^1^Institute of Innovation and Entrepreneurship, Zhejiang Chinese Medical University, Hangzhou, China; ^2^School of Marxism, Zhejiang University, Hangzhou, China

**Keywords:** entrepreneurial psychology, causal attribution theory, entrepreneurship education, new venture management strategies, college student

## Abstract

With the rapid development of information technology, the society’s demand for innovative talents has become increasingly prominent. The purpose of this study is to optimize the teaching strategies of entrepreneurship education for college students, further cultivate college students’ entrepreneurial ideas, and promote the formation of entrepreneurial values. The problems existing in entrepreneurship education in colleges and universities are studied based on entrepreneurial psychology and attribution theory. A questionnaire survey is conducted on the problems with a high probability of entrepreneurial failure of college students. The heads of new ventures in Xi’an are selected. Then, 300 questionnaires are distributed, and 209 are returned. The survey results are analyzed using failure attribution and failure learning. Suggestions are provided for management strategies of new ventures. The results show that the Corrected Item-Total Correlation (CITC) value of R^−1^ is 0.65, and the CITC value of R^−2^ is 0.35. In addition, the Kaiser-Meyer-Olkin (KMO) values of entrepreneurial failure attribution and entrepreneurial failure mode are both greater than 0.7, which indicates that the scale of entrepreneurial failure attribution has good validity and can be used for factor analysis. However, the KMO values of entrepreneurial failure attribution and entrepreneurial failure learning model are both greater than 0.7, and the significance of Bartlett sphericity test is 0.00, which indicates that the survey has good validity. The research has practical application and reference value for the cultivation of college students’ innovative and entrepreneurial ability.

## Introduction

With the development of a new technological revolution, innovation has become an important indicator of international competition (Huang-Saad et al., 2020; Jena, 2020; Liu et al., 2021). As an important place for talent training, the entrepreneurship education concept and education model implemented by colleges have a positive impact on the formation of college students’ entrepreneurial values and the success or failure of future entrepreneurship (Alshebami et al., 2020; Hua and Ren, 2020; Mei and Symaco, 2020). The core of innovation and entrepreneurship education is to cultivate college students’ innovative spirit and entrepreneurial ability. Low-intensity physical activity among students leads to vitality, youthfulness, and other positive emotional changes (Li et al., 2019). At present, the innovative and entrepreneurial spirit, entrepreneurial awareness, social responsibility, and practical ability of college students in China need to be enhanced. The performance is that the concept of entrepreneurship and “creating wealth” is the first choice for the value goal of innovation and entrepreneurship. Emphasis is placed on the individual level of innovation and entrepreneurship, showing a degree of utilitarianism and self-discipline. The development of entrepreneurial spirit and comprehensive quality is neglected. Information asymmetry refers specifically to the fact that parties have different information in a transaction (Zhong et al., 2020). Social media has not only changed the way people communicate with friends but also the way providers communicate with consumers (Shen et al., 2019). How colleges and universities can effectively help college students build entrepreneurial awareness and entrepreneurial ability that matches their entrepreneurial goals also requires more energy, manpower, and material resources to be spent on the entrepreneurial education model.

With the rapid development of technology, the source of knowledge is no longer limited to teachers or books. On the contrary, students can acquire different knowledge through the Internet (Ludwig and Macnaghten, 2020). Market competitiveness and sustainable operation of an enterprise are related to the support of its high-tech core technology (Feng et al., 2020). Liang et al. (2021) pointed out that the research on the sustainability evaluation of innovation and entrepreneurship education for clean energy majors in colleges and universities could not only cultivate innovation and entrepreneurship talents for the development of sustainable energy but also provide the reference for the sustainable development of innovation and entrepreneurship education in other majors. Facing the challenging employment situation and the changing labor market, cultivating students’ entrepreneurial intention had attracted important policy considerations in China. Mei et al. (2020) described the background of entrepreneurship education in Chinese colleges and discussed the impact of entrepreneurship education on students’ entrepreneurial intentions. Students from different types of institutions and professional fields had different levels of participation in entrepreneurship education. In addition, the higher the level of entrepreneurship education students received, the stronger the self-efficacy of entrepreneurial decision-making and the stronger the entrepreneurial intention (Chen and Zhang, 2020; Choi and Hwang, 2020; He, 2020). Students’ self-efficacy in entrepreneurial decision-making plays a mediating role between entrepreneurial education and students’ entrepreneurial intention. It is concluded that entrepreneurship education in colleges and universities has played a prominent role in college students’ entrepreneurship.

This paper uses the methods of literature research and questionnaire survey to study the effective entrepreneurship education in colleges and universities and the management strategies of new ventures. The main variables are changes in entrepreneurial psychology and attribution of entrepreneurial failure. A major gap in current research is the lack of a necessary link between changes in entrepreneurial psychology and the attribution of entrepreneurial failure. Failure attribution is applied to the study of the failure of new ventures through the study of entrepreneurial psychology and attribution theory. In addition, a series of data analyses is carried out. This paper can provide a reference for the improvement of the entrepreneurship education model in colleges and universities and the management of new ventures in the future.

## Literature review

Regarding the causal attribution theory, many scholars have conducted related research. Carlson et al. (2018) studied the problem of causal attribution toward strict telecoupling. Causal attribution methods were reviewed and systematically summarized by moving towards strictly distantly coupled causal attribution methods. The findings suggested that rigorous qualitative and quantitative causal attribution was important for formulating reliable telecoupling systems (Carlson et al., 2018). Hamann et al. (2021) studied the role of self-efficacy, attribution habits, and gender in analyzing college students’ success. Specific cognitive variables were queried. Findings showed that female and male students reported different self-efficacy and causal attribution outcomes (Hamann et al., 2021). Brun et al. (2022) analyzed the underlying characteristics of teachers’ academic success or failure attribution. The research hypothesizes that teachers’ perceptions of the reasons behind students’ academic performance change as causality changes. The analysis identified five teachers’ attribution profiles across four causal dimensions. The results showed that the attribution profiles of profile members largely depended on the student’s outcome valence. Therefore, applying causal attribution theory to college students’ entrepreneurship education and entrepreneurial intention evaluation will help to promote the development of innovation and entrepreneurship education (Brun et al., 2022; Cao, 2022). Wang et al. (2021) studied the influence of entrepreneurship education, mentality, and creativity on entrepreneurial intention. The self-administered survey data of college students from Jiangsu and Zhejiang provinces were collected using structural equation modeling technology and software to verify the hypothesis. The results showed that entrepreneurial education, entrepreneurial mentality and creativity had positive and significant effects on entrepreneurial intention. In addition, entrepreneurial self-efficacy played an intermediary role in the influence of entrepreneurial education on entrepreneurial intention (Wang et al., 2021). Li et al. (2020) studied the role of entrepreneurial passion in the development of entrepreneurial self-efficacy under the influence of positive personality. The results suggested that entrepreneurial passion had a significant positive impact on entrepreneurial alertness, entrepreneurial intention self-efficacy and entrepreneurial behavior. In addition, the positive personality had a significant positive moderating effect on the relationship between entrepreneurial intention and entrepreneurial behavior (Li et al., 2020).

In addition, regarding the development of innovation and entrepreneurship education level, Ndofirepi (2020) researched the relationship between entrepreneurship education and entrepreneurial goal intention. The mediating variable was psychological traits. The relationship between the perceived effects of entrepreneurship education and entrepreneurial intentions was predicted. The findings suggested that entrepreneurship education and training could nurture future entrepreneurs (Ndofirepi, 2020). Song et al. (2021) studied the entrepreneurial intention of college students and the mediating role of entrepreneurship education. Factors influencing college graduates’ entrepreneurial intentions were identified through attitudes, perceived behavioral control, and self-efficacy. The results showed that entrepreneurship education partially mediated attitudes and perceived behavioral control but fully mediated self-efficacy (Song et al., 2021). Hattab and Fahmy (2022) studied the influence of college students’ entrepreneurial self-identity construction. They aimed to investigate the relationship between entrepreneurship education and entrepreneurship. The research had practical application value for promoting the development of college students’ entrepreneurship education level (Hattab and Fahmy, 2022). To sum up, entrepreneurship education and college students’ entrepreneurial activities increase in school activities. Schools should focus on educational innovation and cultivating students’ practical ability. The above literature can support the development of innovation and entrepreneurship education.

## Materials and methods

### Entrepreneurial psychology and causal attribution theory

**Figure 1:**
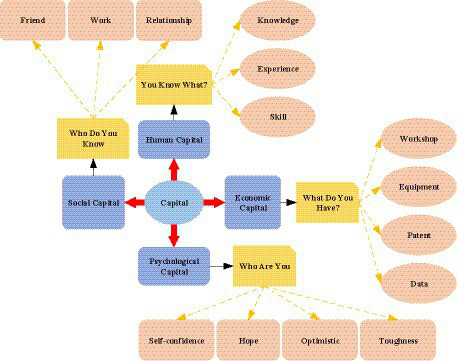
Four capital forms. (Image source: from the literature Luthans et al., 2004).

Entrepreneurial psychology studies the psychological influence factors of entrepreneurs. Research on entrepreneurial psychology helps entrepreneurs adjust their management methods and business models promptly (Obschonka et al., 2020; Su et al., 2020; Wiklund et al., 2020). As an important field of entrepreneurial psychology research, entrepreneurial psychological capital is a specific positive psychological state exhibited by individuals that can affect entrepreneurial-related activities (Chavoushi et al., 2021; Newman et al., 2021; Haddoud et al., 2022). Capital is divided into economic capital, social capital, and human capital according to the development form. Economic capital refers to tangible assets such as money and real estate owned by people, while human capital refers to the comprehensive individual ability and knowledge cognitive ability. The formation of these abilities comes from the education and living environment received. Social capital generally refers to the social environment, interpersonal circle, and other trust resources of individuals. With the development of society, it is found that in addition to these kinds of capital, the psychological strength of enterprise leaders and employees is the core element of enterprise competitiveness. Therefore, the concept of psychological capital has been developed, emphasizing the impact of the individual positive psychological state on the success of entrepreneurship (Gao, 2020; Geng et al., 2021; To et al., 2021). The specific frame structure of the study is shown in Figure 1.

**Figure 2:**
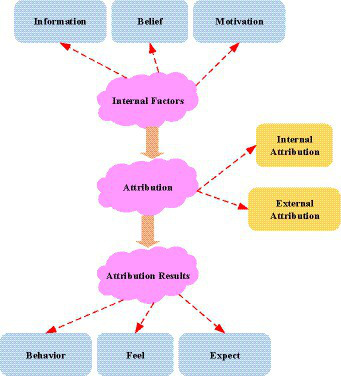
The causal attribution process. (Image source: from the Internet).

From Figure 1, The four main forms of capital are human capital, social capital, economic capital, and psychological capital. Among them, social capital includes friends and social relations. Human capital includes knowledge, experience, and lessons. Economic capital includes workshop equipment and patents. Psychological capital includes attitudes of hope, optimism, and self-confidence. Unlike the psychological capital in Figure 1, entrepreneurial psychological capital is a specific positive psychological state that affects entrepreneurial-related activities. The concept of entrepreneurial psychological capital comes from psychological capital, which is the application of psychological capital in entrepreneurship-related activities. Attribution theory is a set of methods that describe how social perceivers use information to arrive at causal explanations. Part of the reason people keep making causal analyses is that they try to explain society as a whole (DeVellis et al., 1991). People are used to two types of attribution: personality attribution and event attribution. They are also called internal attribution and external attribution. People use “event attribution” to explain their behavior and “personality attribution” to explain the behavior of others. “Personality attribution” attributes the motivation behind certain actions to the personality. “Event attribution” is attributing the motivation behind certain actions to the event itself. The ultimate purpose of attribution is to predict and evaluate people’s behavior to control the environment and behavior. This process of seeking practical causality is the process of attribution. The “causal attribution” process is used for structural modeling and standard judgment. Figure 2 shows the structure of the specific process and model establishment.

From Figure 2, if a student consistently fails and attributes the failure to internal, stable, and uncontrollable factors, he develops a self-feeling of learned helplessness. Learned helplessness is a depressive state experienced by individuals when they feel that no matter what they do, their life events will not be affected. It is believed that there will be no change in this area, and the psychological negativity may continue.

### Attribution analysis of the entrepreneurial failure of new ventures for entrepreneurship education in colleges

In the process of implementing entrepreneurship education, colleges need to continue to refine and classify the implementation of entrepreneurship education. Entrepreneurship education is a way of thinking and behavior that needs to permeate students’ daily education (Ghafar, 2020; Iwu et al., 2021; Sansone et al., 2021). This “entrepreneurial spirit” is exactly what the school’s entrepreneurship education aims to achieve. On the one hand, the improvement and development of entrepreneurship education in China will take time. On the other hand, this kind of education also needs to stimulate the creative spirit of students subtly, so there are many obstacles in the process of realization. After graduation, college students need support from many aspects, such as entrepreneurial environment, capital, and business model. Many factors will affect the ultimate success or failure of entrepreneurship (Aadland and Aaboen, 2020; Cui et al., 2021). From the perspective of entrepreneurial failure, college students’ entrepreneurial dilemma is studied in this paper. A theoretical model of failure attribution for new ventures is built by combining causal attribution theory and failure learning theory. The reasons for the failure of existing new ventures are investigated and analyzed through questionnaires. Entrepreneurial failure attribution can reflect the entrepreneur’s acceptance level of the impact of entrepreneurial failure and understanding the reasons for failure.

**Figure 3:**
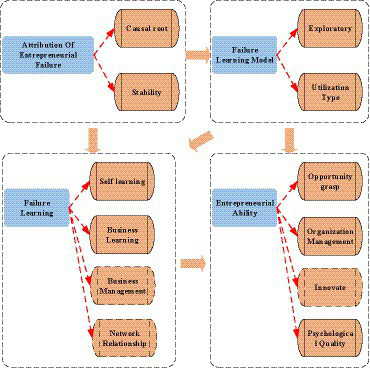
Theoretical model. (Image source: Author’s drawing).

According to signaling theory, entrepreneurial failure can signal entrepreneurs to reflect on their attitudes and behaviors. Entrepreneurs reflect on the entrepreneurial process in the order of releasing signals, transmitting signals, and receiving signals. In the signal release stage, there are also subjective reflections of entrepreneurs in addition to the objective losses of entrepreneurs. In the signaling phase, the founders of the new ventures make attributions for the failure of the new startup. The signal receiving stage is the follow-up behavior of entrepreneurial failure, such as secondary entrepreneurship. Entrepreneurial ability is a test standard for measuring the quality of the signal received by re-entrepreneurship. The attribution of entrepreneurial failure is analyzed based on causal attribution and failure learning theory. Then, the entrepreneurial ability of college students is affected by failure. The frame structure of the theoretical model is shown in Figure 3.

There is generally more than one reason for entrepreneurial failure. Failure attribution is a tool for entrepreneurs to characterize the failure of this venture. Entrepreneurs’ attribution explanation will be significant to developing subsequent entrepreneurial activities. Efficient entrepreneurs use positive attribution to deal with failure and accumulate experience after failure. Therefore, hypothesis H1 is proposed.

*H1:* Attribution of entrepreneurial failure positively impacts entrepreneurial ability.

The attribution of entrepreneurial failure can be divided into internal and external causes from the dimension of causal origin. More emphasis is placed on subjective internal causes in attributing entrepreneurial failure. More focus is placed on self-reflection in the learning process. More emphasis is placed on external environmental factors in attributing entrepreneurial failure. More focus will be placed on adjusting the state and starting a new business in the learning process. From the stability dimension, the attribution of entrepreneurial failure is divided into stable and unstable factors. If it is attributed to unstable factors, entrepreneurs believe such factors will not appear next time. Then, there will be no positive impact on personal ability.

For failure learning, entrepreneurs’ attribution of entrepreneurial failure to their factors is conducive to entrepreneurial learning after failure. However, if entrepreneurs choose external attribution, they tend to ignore their problems and cannot actively obtain industry experience. Therefore, hypothesis H2 is proposed.

*H2:* Entrepreneurial failure attribution positively affects failure learning.

Entrepreneurial failure is an opportunity for entrepreneurs to discover the internal problems of the enterprise and the potential problems of comprehensive personal quality. In failure learning, entrepreneurs not only enrich the knowledge reserve of failure scenarios but also improve their cognitive level by analyzing the reasons for failure. Besides, the entrepreneur’s learning from the failed entrepreneurial experience in the past can enhance the entrepreneur’s opportunity identification ability. Therefore, hypothesis H3 is proposed.

*H3:* Learning from failure positively affects entrepreneurial ability.

If entrepreneurs want to improve their entrepreneurial ability, they not only need to identify internal and external resources that can be used but also learn from key events, such as previous entrepreneurial failure experiences. Entrepreneurs can obtain relevant knowledge and information, thereby enhancing their entrepreneurial ability by mining the value of failure experience. Therefore, the following hypotheses are proposed.

*H4:* The failure learning model mediates the effect of entrepreneurial failure attribution on entrepreneurial ability.

*H5:* The failure learning model mediates the effect of entrepreneurial failure attribution on the content of failure learning.

*H6:* Failure learning content mediates the effect of entrepreneurial failure attribution on entrepreneurial ability.

Given the above hypotheses, this paper selects the university student entrepreneurs in the start-up stage of new ventures in Xi’an from 2019 to 2021 as the research object. There are two conditions. The company has been established for less than 3 years and has obvious entrepreneurial failure experience. The sample data is collected using a questionnaire survey. Questionnaires are distributed in two ways: the website of Questionnaire Star and the on-site distribution and recycling of entrepreneurial bases. Seven sub-questions are designed for the two dimensions of causal origin and attribution stability. Table 1 reveals the specific content.

**Table 1:** Entrepreneurial failure attribution measurement problem items.

Variable	Content
Causal origin	R-1 The reason for the failure is mainly their problems. R-2 They do not go all out. R-3 Someone should be held responsible for the failure.
Stability	F-1 Failure has been expected. F-2 The main influencing factors of failure are formed over a long period. F-3 The main factors affecting failure are inherent. F-4 The main influencing factors of failure are controllable.

The content design of the failure learning model scale is shown in Table 2.

**Table 2:** Failed learning mode measurement problem items.

Variable	Content
Utilization	U-1 Learn ways to adjust product issues.
	U-2 Find a conservative approach to business development.
	U-3 Find a mature approach to product development.
	U-4 Learn from competitors.
Exploratory	E-1 Find different items.
	E-2 Find projects to be verified.
	E-3 Find risky projects.
	E-4 Develop new entrepreneurial projects.

Before the formal questionnaire, a small sample questionnaire test is conducted to eliminate invalid items. The content of the small sample questionnaire is shown in Tables 1, 2, including seven items for the measurement of entrepreneurial failure attribution and eight items for the failure learning pattern scale. The small sample questionnaire collection scope selects new ventures in the start-up stage as of December 31, 2021. The criteria for selecting new ventures are that they have been established within 3 years. The questionnaire respondents are business leaders and have obvious entrepreneurial failure experiences. Besides, 100 questionnaires are distributed in the small sample test, and 77 valid questionnaires are recovered after invalid questionnaires are excluded. Cronbach’s Alpha value is selected for the reliability test. If the value exceeds 0.8, it is considered very credible. The validity test selects Kaiser-Meyer-Olkin (KMO) and Bartlett test to judge whether factor analysis can be performed, and KMO > 0.7 needs to be satisfied. Then, it is proved that the questionnaire has good validity. The KMO test statistic is an index used to compare simple correlation coefficients and partial correlation coefficients between variables and is mainly used in factor analysis of multivariate statistics.

Unclear areas of sample data are identified through survey descriptions. Unqualified questions R^−2^ and F-3 are eliminated, leaving 13 questions for the formal questionnaire survey. Like the small sample test, new ventures are selected in Xi’an. The online questionnaire will share links to entrepreneurship-related communities for collection. Entrepreneurship base inspections are conducted in colleges and universities. Also, 300 questionnaires are distributed, and 209 valid questionnaires are recovered. Then, descriptive statistics and reliability and validity analysis are performed. Sample data are analyzed and processed with SPSS 21.0 software. Two factors are extracted from the independent variable attribution of entrepreneurial failure, and exploratory factor analysis is performed by principal component analysis (Brown, 2006; Doornik and Hansen, 2008; Streiner and Norman, 2008; Hair et al., 2009; Lorenzo-Seva et al., 2011; Reichenheim et al., 2011; Osborne, 2015).

## Results and discussion

The Corrected Item-Total Correlation (CITC) value and reliability test results of the small sample scale are as follows.

**Figure 4:**
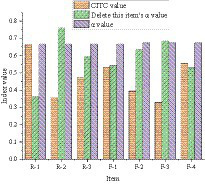
CITC value and reliability analysis of entrepreneurial failure attribution.

The CITC value and reliability test of entrepreneurial failure attribution are shown in Figure 4. Figure 4 manifests that the overall Cronbach’s alpha value of R-1 ~ R-3 for the causal attribution of entrepreneurial failure is 0.673. The overall Cronbach’s alpha value of items F-1 ~ F-4 is 0.666. The CITC value for the R-2 item of causal attribution of entrepreneurial failure is 0.358. The Cronbach’s α value after deletion has increased from 0.673 to 0.760, so this item needs to be deleted. The CITC value of the F-3 item is 0.329, and the Cronbach’s α value after the deletion is increased from 0.666 to 0.685, so this item also needs to be deleted.

**Figure 5:**
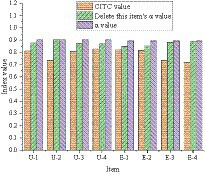
Reliability analysis of entrepreneurial failure learning model.

Figure 5 displays the reliability analysis results of the entrepreneurial failure learning model. From Figure 5, the overall Cronbach’s α value of item U-1 ~ U-4 is 0.909, and the overall Cronbach’s α value of item E-1 ~ E-4 is 0.897. The CITC value and the Cronbach’s α value after deleting the item did not change much, so it’s unnecessary to delete the item.

The results of the validity analysis are shown in Tables 3, 4. From Tables 3, 4, the KMO values of entrepreneurial failure attribution and entrepreneurial failure mode are both greater than 0.7, indicating that the validity of the entrepreneurial failure attribution scale is good, and factor analysis can be carried out.

**Table 3:** KMO and Bartlett test results of entrepreneurial failure attribution.

KMO	0.768	
Bartlett text	Approximate chi-square	105.678
	df	10
	Sig.	0.000

**Table 4:** KMO and Bartlett test results of entrepreneurial failure mode.

KMO	0.864	
Bartlett text	Approximate chi-square	500.812
	df	29
	Sig.	0.000

**Figure 6:**
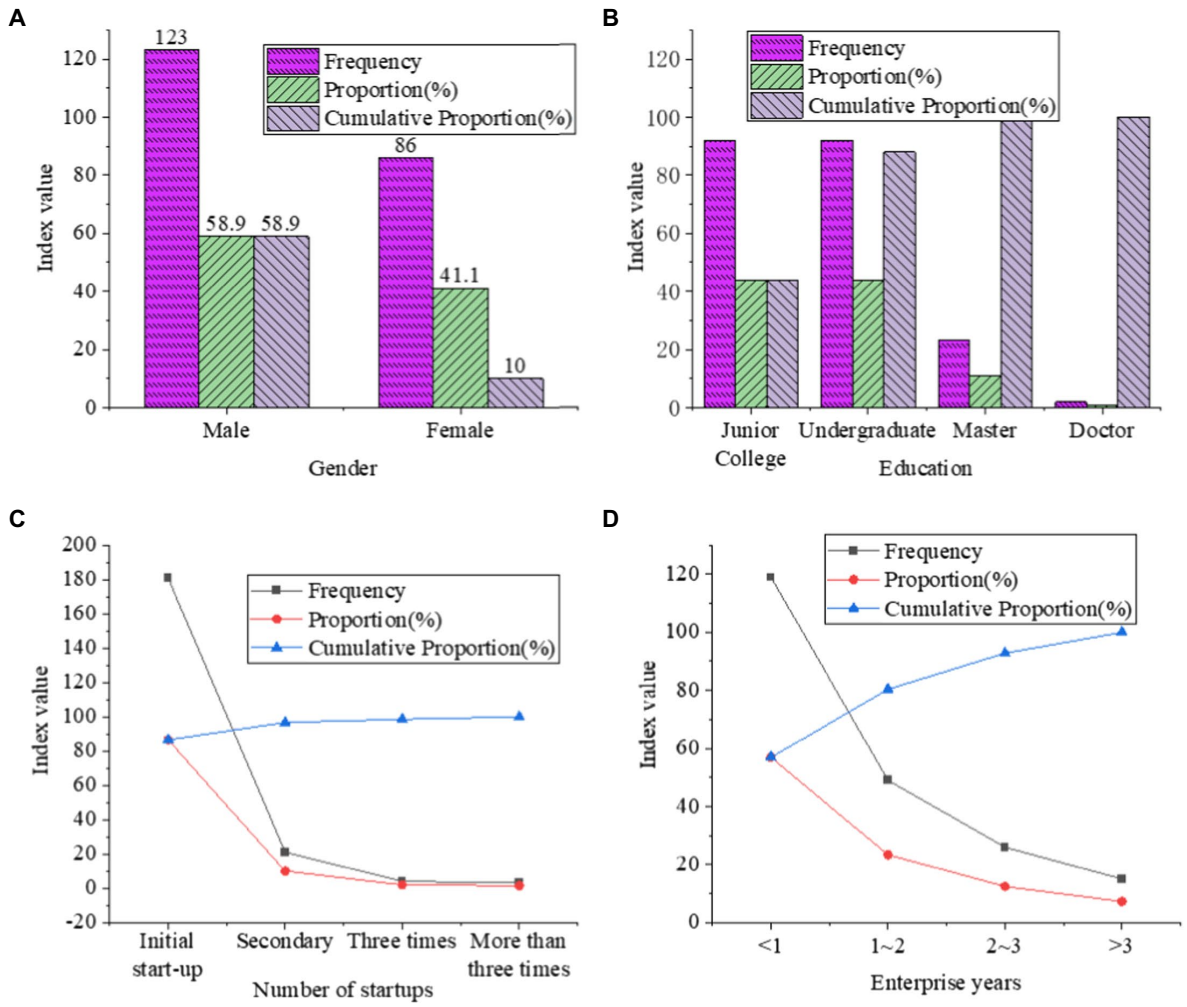
Distribution of financing needs of small and medium-sized enterprises in Jiangsu Province from 2020 to 2021 (**(A)** gender statistics of entrepreneurs; **(B)** educational statistics; **(C)** number of entrepreneurship statistics; **(D)** statistics of entrepreneurial years).

The descriptive statistical results of the survey of the basic information of the respondents are revealed in Figure 6. Figure 6 implies that the proportion of male entrepreneurs in the survey sample is higher than that of females. Both college and undergraduate students account for 44%. Most entrepreneurs are first-time entrepreneurs, accounting for 86.6%. Only 1.9% of the people started businesses more than three times. The number of companies that have been in business for less than 1 year is the largest, accounting for 56.9%.

According to the enterprise life cycle theory, entrepreneurial failure occurs more frequently than entrepreneurial success. The basic data results of the survey generally meet the enterprise life development curve from the perspective of entrepreneurial failure.

**Figure 7:**
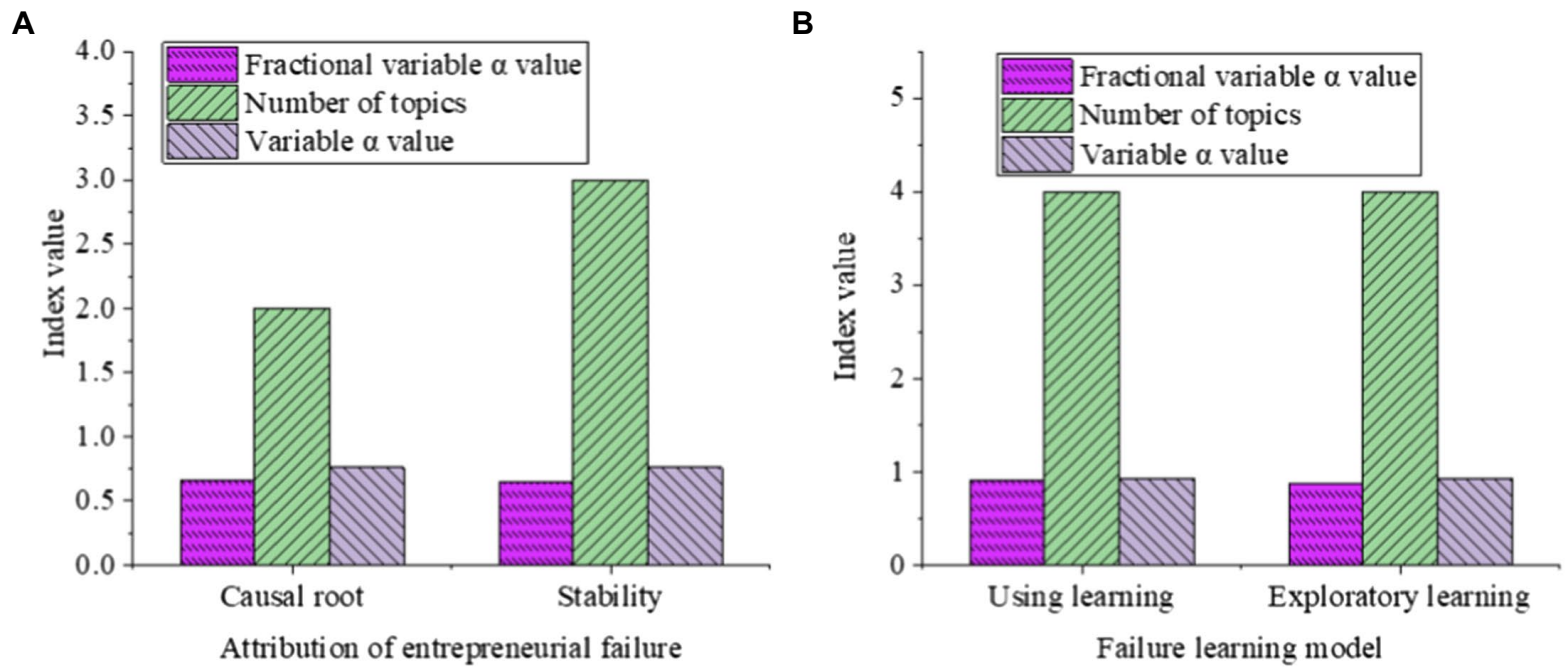
Reliability analysis of each variable (**(A)** entrepreneurial failure attribution scale; **(B)** entrepreneurial failure learning model scale).

**Figure 8:**
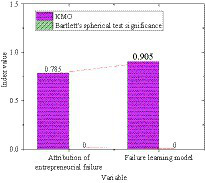
KMO and Bartlett sphericity test results for each variable and the whole.

SPSS21.0 software is used to analyze the reliability and validity of the entrepreneurial failure attribution scale and the entrepreneurial failure learning model scale. Figures 7, 8 display the results. From Figure 7, the Cronbach’s α value of all sub-variables of the entrepreneurial failure attribution scale and the entrepreneurial failure learning model scale is greater than 0.6, and the overall Cronbach’s α value of the questionnaire is 0.985, which is greater than 0.9, indicating that the reliability of the survey is good. From Figure 8, the KMO values of entrepreneurial failure attribution and entrepreneurial failure learning model are both greater than 0.7, and the significance of the Bartlett sphericity test is 0.00, indicating that the survey has good validity.

**Figure 9:**
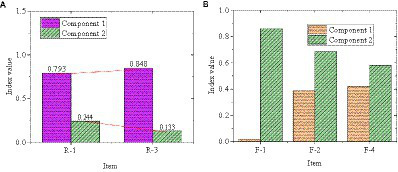
Rotation component matrix of entrepreneurial failure attribution (**(A)** causal root variable; **(B)** stability variable).

**Figure 10:**
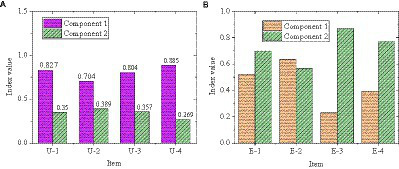
Rotating component matrix of failure learning mode (**(A)** utilization learning; **(B)** exploratory learning).

The loading matrix of principal component analysis is shown in Figure 9. From Figure 9, the common factors one and two are in complete agreement with the two dimensions of entrepreneurial failure attribution, and the factor loadings are all above 0.5, indicating that the dimension division of the scale is reasonable. Two factors are extracted from the variable failure learning mode to obtain the factor load matrix, as revealed in Figure 10. From Figure 10, the common factor two explains the variable of exploratory learning and has a high correlation with the three items E-1, E-3, and E-4. Item E-2 is not on the same factor level as E-1, E-3, and E-4.

The values of the factor loading coefficients of the above two scales are shown in Tables 5, 6.

**Table 5:** Loading coefficient values of entrepreneurial failure attribution factors.

Factor name	Item	Factor loading coefficient
Causal root	R-1	0.820
	R-3	0.903
Attribution stability	F-1	0.723
	F-2	0.689
	F-4	0.816

**Table 6:** Loading coefficient values of failure learning mode factor.

Factor name	Item	Factor loading coefficient
Utilization	U-1	0.798
	U-2	0.723
	U-3	0.800
	U-4	0.884
Exploratory	E-1	0.789
	E-2	0.761
	E-3	0.770
	E-4	0.830

The survey results show that the above hypothesis is verified. The entrepreneurial ability of college students is positively affected by the failure attribution of new ventures and learning from failure. Causal attribution affects the content of failure learning through failure learning models. In addition, causal attribution also directly affects the content of failure learning. Stability affects the content of failure learning entirely through the failure learning model. The failure learning mode affects the entrepreneurial ability of college students through the content of failure learning. Then, the failure learning model loses the mediating effect of entrepreneurial failure attribution on entrepreneurial ability. It is concluded that the effects of failure learning mode and content on failure learning are mutual. The attribution of entrepreneurial failure will be mediated by learning from failure in the process of affecting the entrepreneurial ability of college students. The results of this paper are compared with those of previous literature. Zhou (2021) studied the issues related to the cultivation of innovation and entrepreneurship ability of adult college students. It was found that there were many problems in the cultivation of innovative and entrepreneurial ability of adult college students in the Internet age, such as lack of talents, low entrepreneurial rate and success rate, imperfect school-enterprise cooperation system and mechanism, and unbalanced willingness of both parties. Countermeasures were put forward from four aspects to provide a useful reference for cultivating the innovation and entrepreneurship ability of adult college students in the Internet era (Zhou, 2021). Wang et al. (2022) studied the practical role of innovation and entrepreneurship education for college students in the new era. The results showed that the development of all walks of life was slow, and the number of jobs decreased in recent years. With the expanding group of college graduates, many graduates could not find ideal jobs (Wang et al., 2022). Therefore, the government and schools should strengthen entrepreneurship training and encourage college students to carry out innovation and entrepreneurship development.

## New venture management strategies

Numerous new ventures have risen rapidly and continued to grow, and they are gradually occupying an important position in China’s economic development. However, new ventures often have many management problems, and the failure rate of entrepreneurship is high, so it is necessary to adjust the management strategy of the enterprise in time. For individual business managers, starting a business is not an easy task. New ventures are small companies when they are first established. Both products and teams are immature, and people need to adapt. Therefore, a new venture needs a system that suits it. The establishment of an efficient team is not created by a perfect system. In the early stage of the business, the boss should communicate directly with all partners in the team to reduce bureaucracy and the cost of communication. What a company needs in its early days is flexibility, creativity, and creativity. Everyone adapts to diverse roles, and everyone clearly understands that they are the owners and “leaders” of the company to achieve common goals.

An excellent enterprise culture will involve four aspects: entrepreneurial spirit, transcendence spirit, youthful vitality, and a sense of achievement regardless of the specific spiritual characteristics and cultural inclinations of a corporate soul. These four aspects are concentrated in a person, which are manifested as non-conformity, innovation, self-transcendence, inexhaustible vitality, non-stop curiosity, and a sense of achievement to bring spiritual inspiration. The most fundamental is continuous innovation. Only by sticking to this can one bring transcendence, vitality, and a sense of achievement. If these four spirits can be formed and maintained, enterprises can dominate themselves without the interference of the environment. They are far ahead when they are prosperous, and they also shine through difficulties and move forward bravely when they are in a recession. Similarly, the foundation of these four spirits of an enterprise is continuous innovation. However, an enterprise is made up of employees. Enterprises need to focus on the similarities between enterprises and people, form a community between enterprises and people, and cultivate and manage innovation to maintain the driving force of continuous innovation.

## Conclusion

Innovation and entrepreneurship education in colleges and universities aims to cultivate talents with basic entrepreneurial qualities and innovative personalities. It cultivates college students’ entrepreneurial awareness and innovative spirit. The cultivation of innovative thinking and the formation of entrepreneurial skills are carried out in stages and at different levels. Real entrepreneurship education is teachers guide innovation and teach students to master creative thinking. If the innovative thinking of students can be cultivated, the quality of Chinese youth will be improved. In addition, the success rate of college students’ entrepreneurship will also increase. This paper applies entrepreneurial psychology and attribution theory to the improvement of innovative models of entrepreneurial education. For the high failure rate of college students’ entrepreneurial enterprises, the questionnaire is investigated and analyzed using failure attribution. It is necessary to consider the management strategies of new ventures based on the study’s results. In short, college students’ innovation and entrepreneurial ability can be greatly improved by learning the lessons of entrepreneurial failure. The purpose of university entrepreneurship education is to propose new things based on original ideas, theories, and systems to achieve positive social benefits. However, there are some limitations, such as the small scale of the questionnaire and the large randomness of the sample, which may lead to errors in the survey results. Therefore, the research scope should be expanded in future research. The current situation of college students’ entrepreneurship education should be studied in depth to optimize the enterprise risk management strategy and promote the improvement of college students’ innovation and entrepreneurship levels.

## Data availability statement

The raw data supporting the conclusions of this article will be made available by the authors, without undue reservation.

## Ethics statement

The studies involving human participants were reviewed and approved by Zhejiang Chinese Medical University Ethics Committee. The patients/participants provided their written informed consent to participate in this study. Written informed consent was obtained from the individual(s) for the publication of any potentially identifiable images or data included in this article.

## Author contributions

All authors listed have made a substantial, direct, and intellectual contribution to the work and approved it for publication.

## Conflict of interest

The authors declare that the research was conducted in the absence of any commercial or financial relationships that could be construed as a potential conflict of interest.

## Publisher’s note

All claims expressed in this article are solely those of the authors and do not necessarily represent those of their affiliated organizations, or those of the publisher, the editors and the reviewers. Any product that may be evaluated in this article, or claim that may be made by its manufacturer, is not guaranteed or endorsed by the publisher.
